# Supplementary material for: Comparison of Phytochemical Composition and Untargeted Metabolomic Analysis of an Extract from Cnidoscolus aconitifolius (Mill.) I. I. Johnst and Porophyllum ruderale (Jacq.) Cass. and Biological Cytotoxic and Antiproliferative Activity In Vitro
Source: Plants (Basel). 2023 May 15;12(10):1987. doi: 10.3390/plants12101987 (PMC10222540; doi:10.3390/plants12101987)
Supplement: Supplementary file 1 [file plants-12-01987-s001.zip › plants-2360447-supplementary.pdf]

plants

MDPI

Supplementary Files

Comparison of Phytochemical Composition and Untargeted Metabolomic Analysis of an Extract from *Cnidoscolus aconitifolius* (Mill.) I. I. Johnst and *Porophyllum ruderale* (Jacq.) Cass. and Biological Cytotoxic and Antiproliferative Activity In Vitro

Ángel Félix Vargas-Madriz [1,†], Ivan Luzardo-Ocampo [2,3,†], Ulisses Moreno-Celis [1], Octavio Roldán-Padrón [1], Jorge Luis Chávez-Servín [1], Haydé A. Vergara-Castañeda [4], Mónica Martínez-Pacheco [1,5], Carmen Mejía [1], Teresa García-Gasca [1] and Aarón Kuri-García [1,*]

[1] Department of Cell and Molecular Biology, School of Natural Sciences, Universidad Autonoma de Queretaro, Querétaro 76230, Mexico; angel.vargas@uaq.mx (Á.F.V.-M.); ulisses.moreno@uaq.mx (U.M.-C.); octavio.roldan@uaq.mx (O.R.-P.); jorge.chavez@uaq.mx (J.L.C.-S.); monicalmp@iibiomedicas.unam.mx (M.M.-P.); maria.c.mejia@uaq.mx (C.M.); tggasca@uaq.mx (T.G.-G.)
[2] Tecnologico de Monterrey, The Institute for Obesity Research, Ave. Eugenio Garza Sada 2501 Sur, Monterrey 64849, Mexico; ivan.8907@gmail.com
[3] Tecnologico de Monterrey, School of Engineering and Science, Campus Guadalajara, Av. General Ramon Corona 2514, Zapopan 45201, Mexico
[4] Advanced Biomedical Research Center, School of Medicine, Universidad Autonoma de Queretaro, Querétaro 76010, Mexico; hayde.vergara@uaq.mx
[5] Laboratorio de Biomedicina Interdisciplinaria, School of Natural Sciences, Universidad Autonoma de Queretaro, Querétaro 76230, Mexico
* Correspondence: aaron.kuri@uaq.mx
† These authors contributed equally to this manuscript.

Citation: Vargas-Madriz, Á.F.; Luzardo-Ocampo, I.; Moreno-Celis, U.; Roldán-Padrón, O.; Chávez-Servín, J.L.; Vergara-Castañeda, H.A.; Martínez-Pacheco, M.; Mejía, C.; García-Gasca, T.; Kuri-García, A. Comparison of Phytochemical Composition and Untargeted Metabolomic Analysis of an Extract from *Cnidoscolus aconitifolius* (Mill.) I. I. Johnst and *Porophyllum ruderale* (Jacq.) Cass. and Biological Cytotoxic and Antiproliferative Activity In Vitro. *Plants* **2023**, *12*, 1987. https://doi.org/10.3390/plants12101987

Academic Editors: Irma Podolak and Agnieszka Galanty

Received: 6 April 2023
Revised: 5 May 2023
Accepted: 11 May 2023
Published: 15 May 2023

Copyright: © 2023 by the authors. Licensee MDPI, Basel, Switzerland. This article is an open access article distributed under the terms and conditions of the Creative Commons Attribution (CC BY) license (https://creativecommons.org/licenses/by/4.0/).

Supplementary Table S1 Polyphenol, selected acids, and other compounds profile of CA and PR extracts by UPLC-DAD-QToF/MS-ESI.

Ionization mode	Compound name	CAPR		Observed RT (min)	Expected mass (Da)	Observed m/z	Mass error (ppm)	Adducts	Fragments
Hydroxycinnamic acids and derivatives									
ESI-	Trans-cinnamic acid	+		6.99	148.0524	148.0523	-0.80	[M-H] -	147.04514, 103.05562, 101.0249
ESI-	*p*-Coumaric acid	+	+	7.38	167.0574	164.0473	-0.20	[M-H] -	163.04002, 119.0505, 117.0539
ESI-	*m*-Coumaric acid	+		7.38	167.0574	164.0473	-0.20	[M-H] -	119.0502, 93.03453, 117.0539
ESI-	Isoferulic acid		+	8.16	194.0579	194.0580	0.10	[M-H] -	135.04484, 134.03711, 133.02948, 132.02168
ESI-	Ferulic/trans ferulic acid	+	+	8.33	194.0579	194.0580	0.40	[M-H] -	149.02437, 175.00417, 134.03858
ESI-	Sinapic acid	+	+	8.36	224.0685	224.0685	0.10	[M-H] -	149.02437, 164.0468, 208.03751, 193.01406
ESI-	Chlorogenic acid		+	10.57	354.0950	354.0954	0.90	[M-H] -	135.04502, 191.05598, 161.02461, 93.03489
ESI+	Caffeic acid	+	+	10.72	180.0422	180.0428	3.1	[M+H] +	163.03961, 89.03928, 135.04386, 145.02879
Benzoic acid derivatives and benzaldehydes									
ESI-	Vanillin	+		7.06	152.0473	152.0472	-0.80	[M-H] -	151.03769, 136.03952, 108.02151
ESI-	Vanillic acid	+		7.05	168.0423	168.0418	-3.0	[M-H] -	108.02151, 152.01139, 153.01921
ESI-	Protocatechuic acid		+	7.80	154.0266	154.0262	-2.70	[M-H] -	109.02928, 108.0216, 81.03493
ESI-	Catechol		+	9.67	110.0368	110.0365	-2.60	[M-H] -	109.02961, 108.02031, 91.01858
ESI-	4-hydroxybenzoic acid	+	+	10.35	138.0316	138.0314	-2.10	[M-H] -	93.03452, 137.02386
ESI-	Syringic acid		+	10.57	198.0528	198.0528	-0.20	[M-H] -	121.02959, 138.03222,

									123.00863, 78.01126
Flavonols									
ESI-	Quercetin-3-*O*-rhamnosyl-galactoside	+	+	8.82	610.1533	610.1525	-1.50	[M-H] -	255.02931, 119.0504, 225.16051, 183.04478, 185.02377
ESI+	Fisetin	+	+	8.91	286.0477	286.0474	-1.20	[M+H] +	287.05489, 288.05914, 213.05544, 137.02369
ESI+	Rutin	+	+	9.08	610.1534	610.1545	1.50	[M+H] +	303.04982, 611.16181, 465.10503
ESI-	Quercetin-3-*O*-galactoside (Hyperoxide)		+	9.21	464.0955	464.0956	0.20	[M-H] -	300.02724, 271.02440, 301.03430, 255.02955, 302.03781
ESI+	Quercetin-7-*O*-glucoside		+	9.30	464.0955	464.0973	4.0	[M+H] +	303.04961, 285.04033, 109.02874, 163.03921
ESI-	Fisetin	+	+	10.20	286.0477	286.0475	-0.80	[M-H] -	285.03988, 286.04314, 163.00325
ESI-	Quercetin	+	+	11.03	302.0426	302.0430	1.2	[M-H] -	301.03492, 178.09841, 151.00237
Flavones									
ESI-	Gallocatechin		+	6.99	306.0739	306.0725	-4.80	[M-H] -	125.02448, 109.02842, 124.01557, 139.08699
ESI-	Epigallocatechin		+	6.99	306.0739	306.0725	-4.80	[M-H] -	165.07467, 125.02448, 109.02842, 139.08699
ESI-	Myricetin		+	7.45	318.0375	318.0379	1.00	[M-H] -	137.02512, 109.02808, 178.02227
ESI-	Apigenin		+	8.40	270.0528	270.0528	-0.20	[M-H] -	149.0454, 269.0455, 151.004
ESI+	Kaempferol-3-*O*-rutinoside (nicotiflorin)	+		8.92	594.1585	594.1586	0.30	[M+H] +	287.05473, 449.11553
ESI+	Luteolin	+		9.51	286.0477	286.0477	0.00	[M+H] +	287.05503, 153.01902, 117.05806
ESI-	Kaempferol-3-*O*-D-glucoside (Astragalin)	+	+	9.67	448.1005	448.1009	0.70	[M-H] -	255.02962, 447.09349,

									284.03251, 227.03474
ESI+	Kaempferol	+	+	9.73	286.0477	286.0481	-1.20	[M+H] $^{+}$	287.05627, 153.02005, 165.01873
ESI+	Morin	+	+	9.77	302.0426	303.0499	-0.20	[M+H] $^{+}$	303.05061, 153.02005, 229.05217
Flavanones									
ESI-	Pinocembrin		+	11.05	256.0736	256.0733	-1.00	[M-H] $^{-}$	255.02989, 227.03493, 151.00418
ESI-	Naringenin	+		11.78	272.0685	272.0684	-0.30	[M-H] $^{-}$	271.06095, 119.05018, 151.00262, 177.01914
Phenylpropanoic acids									
ESI-	3-(2-hydroxyphenyl) propanoic acid	+	+	9.15	166.0629	166.0630	-0.10	[M-H] $^{-}$	165.05578, 147.04505, 119.05045
Other compounds									
ESI-	Glucuronic acid		+	0.76	194.0426	194.0423	-1.70	[M-H] $^{-}$	113.02403, 85.02986, 71.01426, 59.01395
ESI-	Quinic acid	+	+	0.80	192.0634	192.0635	0.40	[M-H] $^{-}$	191.05612, 85.02976, 109.04109, 93.03461
ESI-	Hippuric acid	+		6.98	179.0582	179.0581	-0.50	[M-H] $^{-}$	164.07177, 147.04514, 134.04551
ESI-	Phenylacetic acid	+	+	7.38	136.0524	136.0522	-1.90	[M-H] $^{-}$	135.04485, 134.03748, 107.05043, 136.04749
ESI-	Resveratrol	+	+	9.13	228.0786	228.0784	-0.90	[M-H] $^{-}$	227.03467, 119.05045, 225.16051, 183.04478, 185.02377

CA: *C. aconitifolius* extract; Da: Dalton; ESI: Electrospray ionization; ppm: parts per million; PR: *P. ruderale* extract; RT: Retention time. The symbol (+) indicates the presence of the compound for either CA or PR extracts.

Supplementary Table S2. Interacting amino acids from the *in silico* analysis between protein targets (APC and KRAS) and selected phenolic compounds.

Protein target	Phenolic compounds from CA or PR	Interaction amino acids
APC	Rutin	ALA555, ARG554, ASN515, GLU468, ILE357,PRO354, VAL513
	Epigallocatechin gallate	ALA514, ASN467, GLN473, GLU468, HIS464, ILE357, PRO354, THR518
	Luteolin	ALA514, ASN467, ASP512, ARG463, GLU460, PHE510, VAL513
K-RAS	Rutin	GLU3, GLU76, HIS166, LYS5, LYS104, LYS167, THR74
	Epigallocatechin gallate	ARG73, GLU76, HIS166, LYS5, LYS104, LYS167, PRO110, THR74, VAL103
	Chlorogenic acid	ARG135, GLN131, GLU143, ILE139, ILE142, PHE141

Supplementary Table S3. Free Energy binding values for the three most probable docking sites between quantified phenolic compounds from CA and PR, and target proteins (APC and K-RAS).

Phenolic compounds	Best binding energy for APC (kcal/mol)	Best binding energy for K-RAS (kcal/mol)
Apigenin	-7.60	-7.30
Caffeic acid	-5.90	-5.70
(+)-Catechin	-7.40	-7.50
Chlorogenic acid	-7.30	-7.50
Epicatechin	-7.30	-7.20
Epigallocatechin gallate	-7.90	-7.90
Ferulic acid	-5.80	-5.60
Gallic acid	-5.20	-5.60
Hydroxybenzoic acid	-4.80	-5.30
Hydroxyphenylacetic acid	-5.30	-5.30
Luteolin	-7.80	-7.30
p-Coumaric acid	-5.50	-5.60
Quercetin	-7.80	-7.30
Resveratrol	-6.80	-6.50
Rosmarinic acid	-7.00	-6.90
Rutin	-8.50	-8.70
Sinapic acid	-5.70	-5.90
Vanillin	-4.70	-5.10

Results are expressed as the mean ± SD of three independent measurements in triplicate. Asterisks indicate significant differences ($p<0.05$) by Student's t-test. CA: *C. aconitifolius*; CE: (+)-catechin equivalents; CT: Condensed tannins; GAE: Gallic acid equivalents; LE: Lyophilized extract; PR: *P. ruderale*; TFC: Total flavonoids content; TPC: Total phenolic compounds.

Supplementary Table S4. Participation of each variable on each component from Principal Component Analysis (PCA).

	**PC1**	**PC2**	**PC3**
TPC	-0.17	1.27	0.04
TFC	1.12	-0.23	0.11
CT	1.13	-0.24	0.10
Apigenin	0.59	0.48	0.91
Caffeic acid (CA)	1.11	-0.26	0.09
Chlorogenic acid (CHA)	-0.17	1.27	0.03
p-Coumaric acid (Cou)	-0.16	1.27	0.05
Epicatechin (Epicat)	0.80	0.58	-0.57
Epigallocatechin gallate (Epigal)	0.80	0.58	-0.57
Ferulic acid (FA)	1.11	-0.26	0.09
Gallic acid (GA	-0.14	1.26	0.07
Hydroxybenzoic acid (Hydroxy-B)	0.80	0.58	-0.57
Hydroxyphenylacetic acid (Hydroxy-P)	0.80	0.58	-0.57
Luteolin	0.59	0.48	0.91
Quercetin	0.80	0.58	-0.57
Resveratrol	0.59	0.48	0.91
Rosmarinic acid (RA)	0.59	0.48	0.91
Rutin	0.80	0.58	-0.57
Sinapic acid (SA)	0.80	0.58	-0.57
Vanillin	0.59	0.48	0.91
Live Cells	0.29	-0.47	0.72
Early Apoptosis	0.63	-0.76	0.17
Late Apoptosis	-0.67	0.68	-0.27
Total Apoptosis	0.54	-0.67	-0.74
Viability	1.12	-0.22	0.07
G0	1.11	-0.22	0.12
S	1.06	-0.08	0.11
G2	-0.80	-0.28	-0.14
LDH	-0.91	0.46	-0.23
Apc	0.80	-0.61	-0.75
Kras	-0.08	-0.20	1.05

CT: Condensed tannins; LDH: Lactate dehydrogenase; TFC: Total flavonoid content; TPC: Total phenolic acids.

Supplementary Table S5. Primer sequences for the real-time polymerase chain reaction (qPCR).

Gene	Forward (5′-3′)	Reverse (5′-3′)
Apc	TTGATAGCTACAAATGAGGACCA	ACAAAGTTCCACATGCATTACTG
Kras	TGTGATTTGCCTTCTAGAACAGT	ACACCCTGTCTTGTCTTTGCT

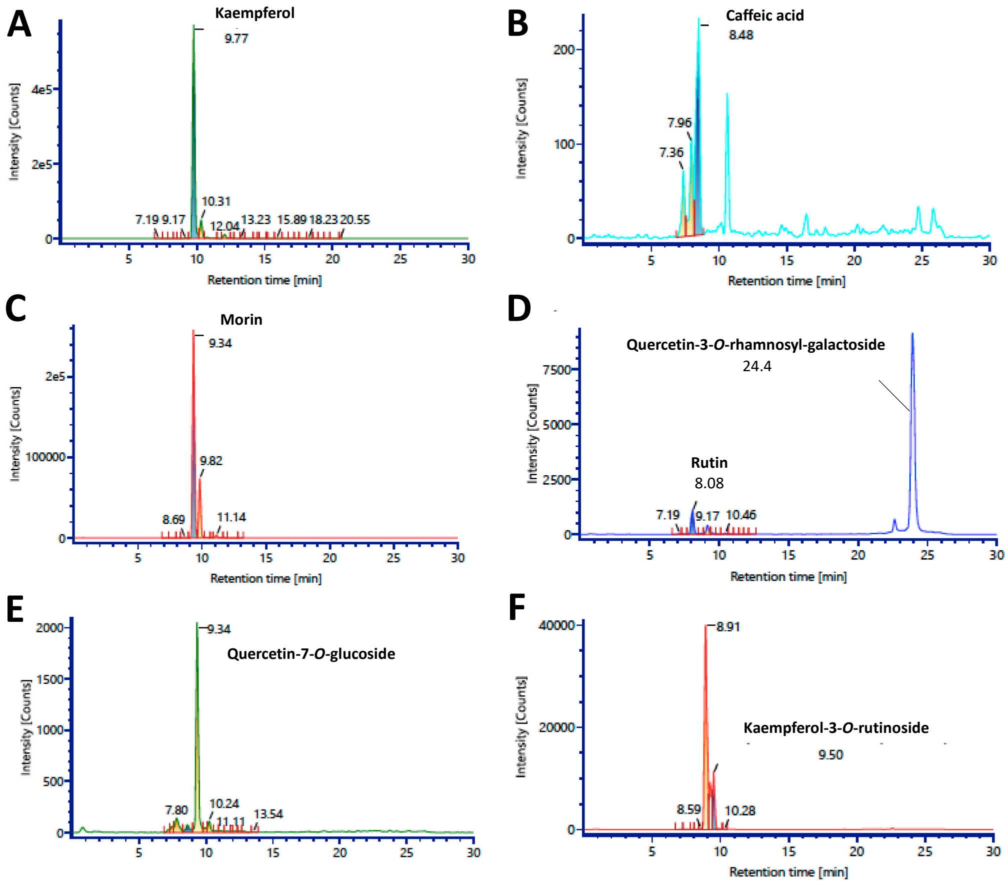

Supplementary Figure S1. Representative pictures from the Mass spectrometry analysis (ESI+) of some of the identified metabolites in CA and PR samples. **(A)** Kaempferol; **(B)** Caffeic acid; **(C)** Morin; **(D)** Quercetin-3-*O*-rhamnosyl-galactoside; **(E)** Quercetin-7-*O*-glucoside; **(F)** Kaempferol-3-*O*-rutinoside.

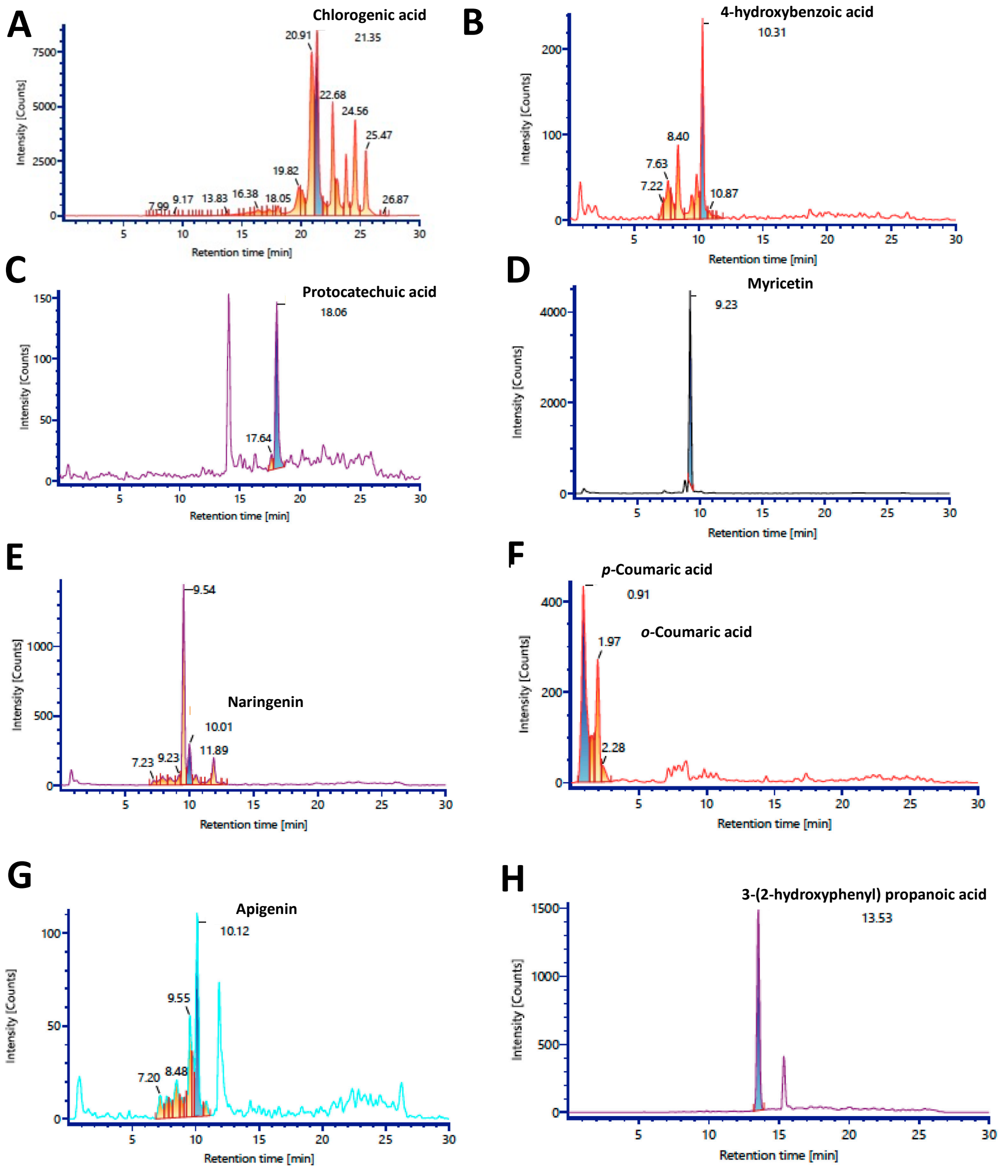

Supplementary Figure S2. Representative pictures from the Mass spectrometry analysis (ESI-) of some of the identified metabolites in CA and PR samples. **(A)** Chlorogenic acid; **(B)** 4-hydroxybenzoic acid; **(C)** Protocatechuic acid; **(D)** Myricetin; **(E)** Naringenin; **(F)** *p*-coumaric and *o*-coumaric acids; **(G)** Apigenin; **(H)** 3-(2-hydroxyphenyl)-propanoic acid.

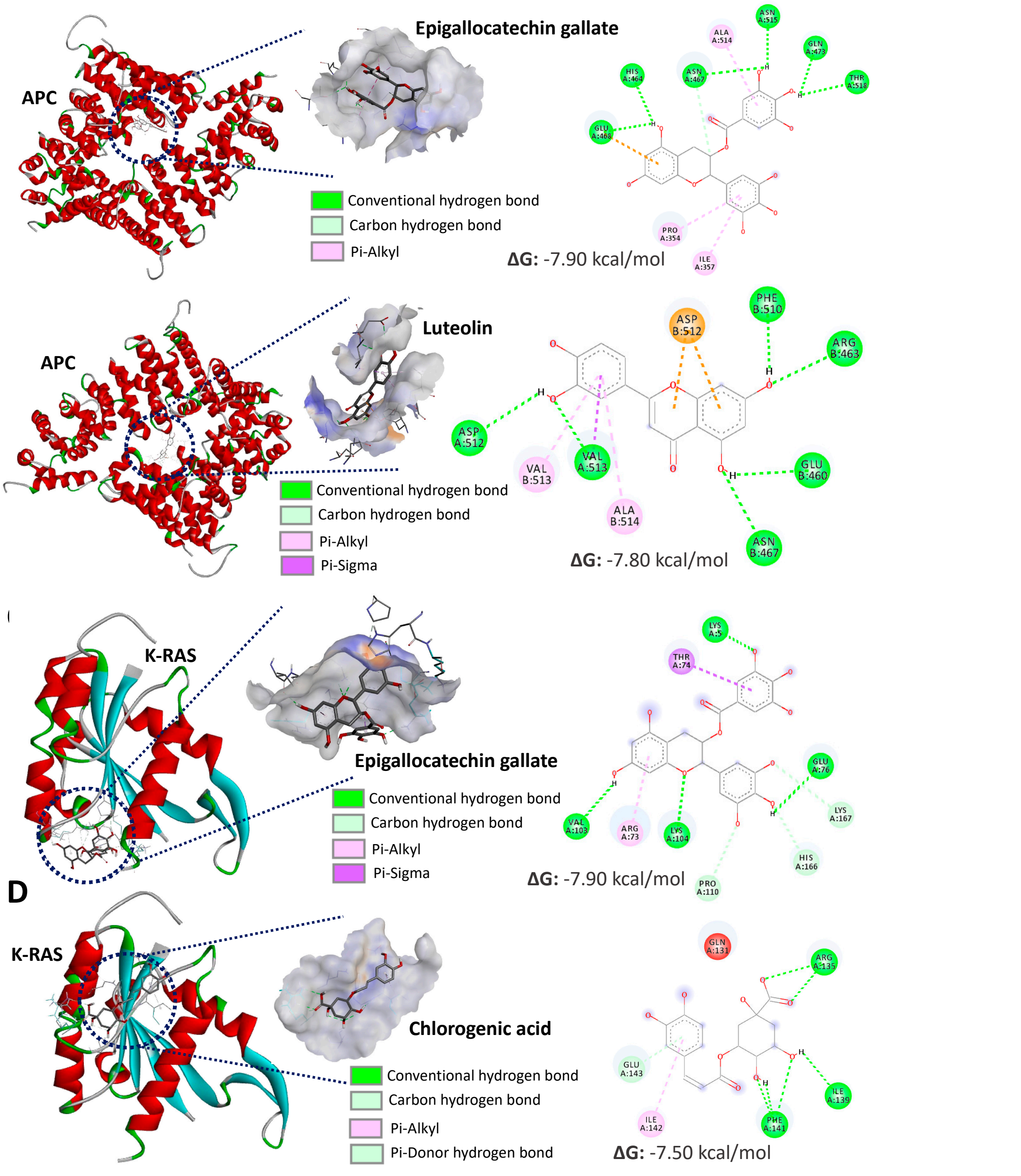

Supplementary Figure S3. *In silico* analysis of the binding affinity between selected phenolics of CA and PR and proteins coded by the assessed genes. Binding affinity between APC protein and (A) Epigallocatechin gallate; (B) Luteolin. Binding affinity between K-RAS protein and (C) Epigallocatechin gallate; (D) Chlorogenic acid. ΔG: Gibbs' energy; CA: *C. aconitifolius*; PR: *P. ruderale.*
